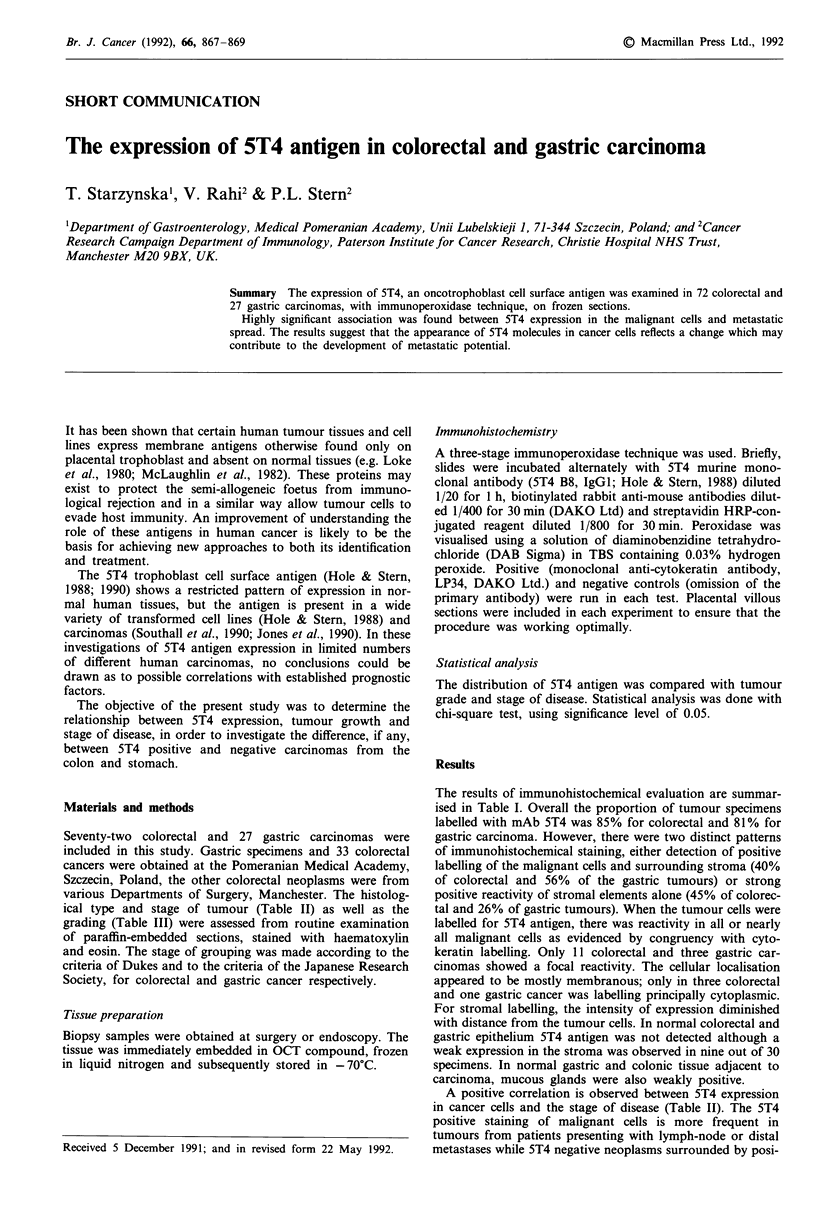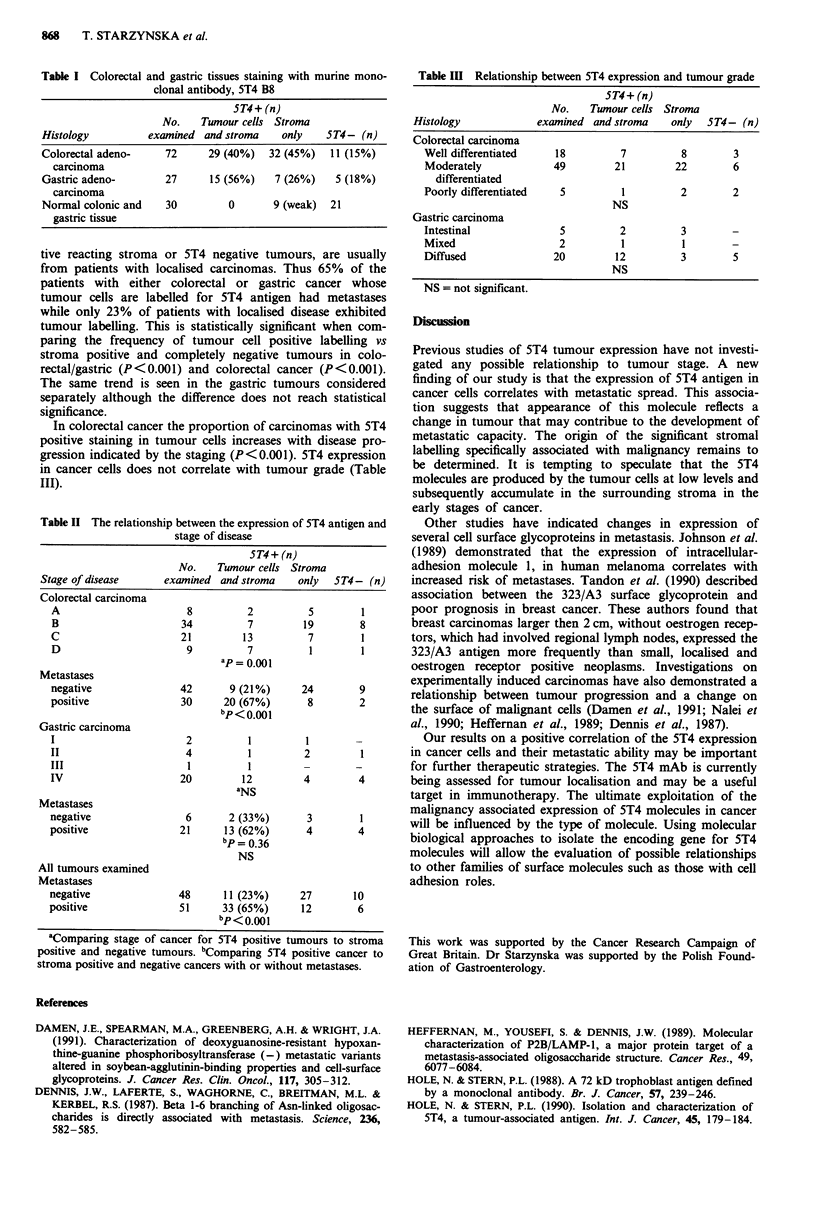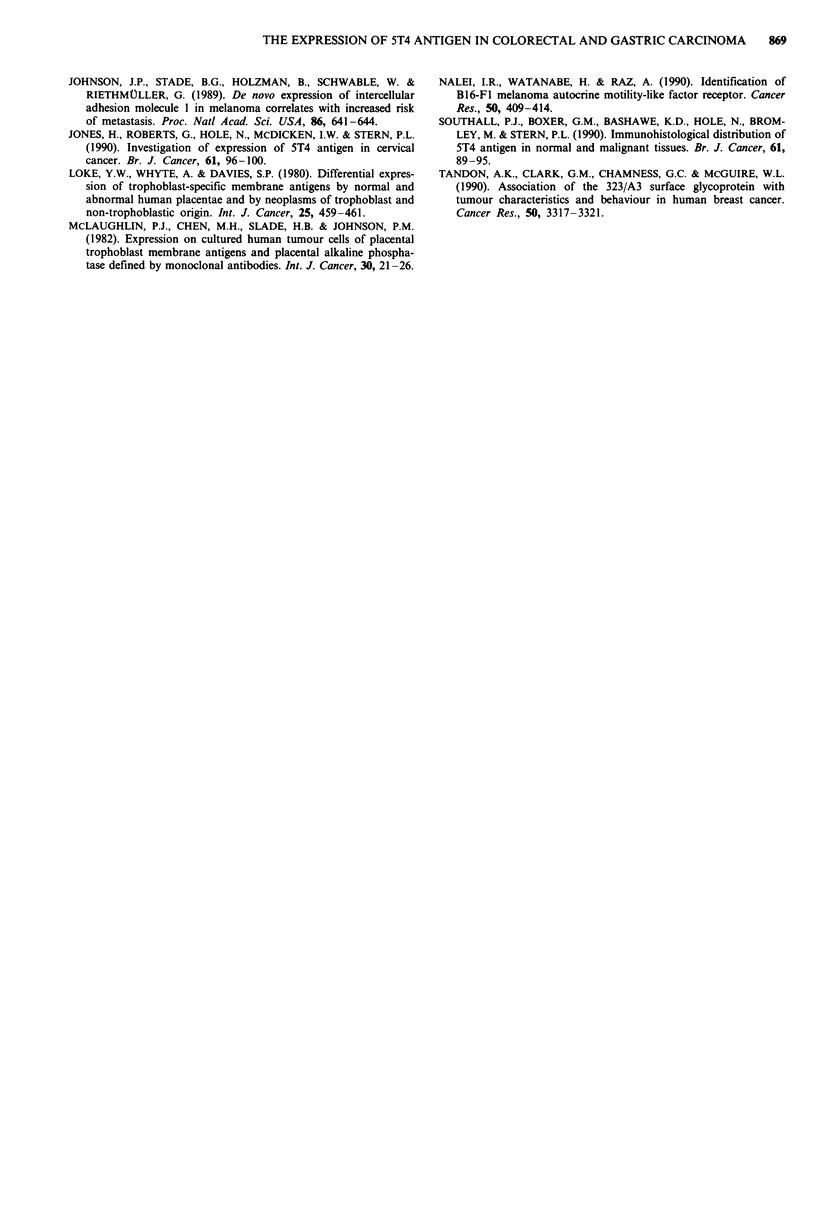# The expression of 5T4 antigen in colorectal and gastric carcinoma.

**DOI:** 10.1038/bjc.1992.375

**Published:** 1992-11

**Authors:** T. Starzynska, V. Rahi, P. L. Stern

**Affiliations:** Department of Gastroenterology, Medical Pomeranian Academy, Szczecin, Poland.

## Abstract

The expression of 5T4, an oncotrophoblast cell surface antigen was examined in 72 colorectal and 27 gastric carcinomas, with immunoperoxidase technique, on frozen sections. Highly significant association was found between 5T4 expression in the malignant cells and metastatic spread. The results suggest that the appearance of 5T4 molecules in cancer cells reflects a change which may contribute to the development of metastatic potential.


Br. J. Cancer (1992), 66, 867-869
© Macmillan Press Ltd., 1992

**SHORT COMMUNICATION**

### The expression of 5T4 antigen in colorectal and gastric carcinoma

T. Starzynska[1], V. Rahi[2] & P.L. Stern[2]

[1]*Department of Gastroenterology, Medical Pomeranian Academy, Unii Lubelskieji 1, 71-344 Szczecin, Poland; and* [2]*Cancer Research Campaign Department of Immunology, Paterson Institute for Cancer Research, Christie Hospital NHS Trust, Manchester M20 9BX, UK.*

**Summary** The expression of 5T4, an oncotrophoblast cell surface antigen was examined in 72 colorectal and 27 gastric carcinomas, with immunoperoxidase technique, on frozen sections.

Highly significant association was found between 5T4 expression in the malignant cells and metastatic spread. The results suggest that the appearance of 5T4 molecules in cancer cells reflects a change which may contribute to the development of metastatic potential.

It has been shown that certain human tumour tissues and cell lines express membrane antigens otherwise found only on placental trophoblast and absent on normal tissues (e.g. Loke *et al.*, 1980; McLaughlin *et al.*, 1982). These proteins may exist to protect the semi-allogeneic foetus from immunological rejection and in a similar way allow tumour cells to evade host immunity. An improvement of understanding the role of these antigens in human cancer is likely to be the basis for achieving new approaches to both its identification and treatment.

The 5T4 trophoblast cell surface antigen (Hole & Stern, 1988; 1990) shows a restricted pattern of expression in normal human tissues, but the antigen is present in a wide variety of transformed cell lines (Hole & Stern, 1988) and carcinomas (Southall *et al.*, 1990; Jones *et al.*, 1990). In these investigations of 5T4 antigen expression in limited numbers of different human carcinomas, no conclusions could be drawn as to possible correlations with established prognostic factors.

The objective of the present study was to determine the relationship between 5T4 expression, tumour growth and stage of disease, in order to investigate the difference, if any, between 5T4 positive and negative carcinomas from the colon and stomach.

### Materials and methods

Seventy-two colorectal and 27 gastric carcinomas were included in this study. Gastric specimens and 33 colorectal cancers were obtained at the Pomeranian Medical Academy, Szczecin, Poland, the other colorectal neoplasms were from various Departments of Surgery, Manchester. The histological type and stage of tumour (Table II) as well as the grading (Table III) were assessed from routine examination of paraffin-embedded sections, stained with haematoxylin and eosin. The stage of grouping was made according to the criteria of Dukes and to the criteria of the Japanese Research Society, for colorectal and gastric cancer respectively.

### *Tissue preparation*

Biopsy samples were obtained at surgery or endoscopy. The tissue was immediately embedded in OCT compound, frozen in liquid nitrogen and subsequently stored in −70°C.

### *Immunohistochemistry*

A three-stage immunoperoxidase technique was used. Briefly, slides were incubated alternately with 5T4 murine monoclonal antibody (5T4 B8, IgG1; Hole & Stern, 1988) diluted 1/20 for 1 h, biotinylated rabbit anti-mouse antibodies diluted 1/400 for 30 min (DAKO Ltd) and streptavidin HRP-conjugated reagent diluted 1/800 for 30 min. Peroxidase was visualised using a solution of diaminobenzidine tetrahydrochloride (DAB Sigma) in TBS containing 0.03% hydrogen peroxide. Positive (monoclonal anti-cytokeratin antibody, LP34, DAKO Ltd.) and negative controls (omission of the primary antibody) were run in each test. Placental villous sections were included in each experiment to ensure that the procedure was working optimally.

### *Statistical analysis*

The distribution of 5T4 antigen was compared with tumour grade and stage of disease. Statistical analysis was done with chi-square test, using significance level of 0.05.

### Results

The results of immunohistochemical evaluation are summarised in Table I. Overall the proportion of tumour specimens labelled with mAb 5T4 was 85% for colorectal and 81% for gastric carcinoma. However, there were two distinct patterns of immunohistochemical staining, either detection of positive labelling of the malignant cells and surrounding stroma (40% of colorectal and 56% of the gastric tumours) or strong positive reactivity of stromal elements alone (45% of colorectal and 26% of gastric tumours). When the tumour cells were labelled for 5T4 antigen, there was reactivity in all or nearly all malignant cells as evidenced by congruency with cytokeratin labelling. Only 11 colorectal and three gastric carcinomas showed a focal reactivity. The cellular localisation appeared to be mostly membranous; only in three colorectal and one gastric cancer was labelling principally cytoplasmic. For stromal labelling, the intensity of expression diminished with distance from the tumour cells. In normal colorectal and gastric epithelium 5T4 antigen was not detected although a weak expression in the stroma was observed in nine out of 30 specimens. In normal gastric and colonic tissue adjacent to carcinoma, mucous glands were also weakly positive.

A positive correlation is observed between 5T4 expression in cancer cells and the stage of disease (Table II). The 5T4 positive staining of malignant cells is more frequent in tumours from patients presenting with lymph-node or distal metastases while 5T4 negative neoplasms surrounded by posi-

Received 5 December 1991; and in revised form 22 May 1992.

**Table I** Colorectal and gastric tissues staining with murine monoclonal antibody, 5T4 B8

| Histology | No. examined | 5T4+ (n) Tumour cells and stroma | Stroma only | 5T4− (n) |
|---|---|---|---|---|
| Colorectal adenocarcinoma | 72 | 29 (40%) | 32 (45%) | 11 (15%) |
| Gastric adenocarcinoma | 27 | 15 (56%) | 7 (26%) | 5 (18%) |
| Normal colonic and gastric tissue | 30 | 0 | 9 (weak) | 21 |

tive reacting stroma or 5T4 negative tumours, are usually from patients with localised carcinomas. Thus 65% of the patients with either colorectal or gastric cancer whose tumour cells are labelled for 5T4 antigen had metastases while only 23% of patients with localised disease exhibited tumour labelling. This is statistically significant when comparing the frequency of tumour cell positive labelling *vs* stroma positive and completely negative tumours in colorectal/gastric ($P<0.001$) and colorectal cancer ($P<0.001$). The same trend is seen in the gastric tumours considered separately although the difference does not reach statistical significance.

In colorectal cancer the proportion of carcinomas with 5T4 positive staining in tumour cells increases with disease progression indicated by the staging ($P<0.001$). 5T4 expression in cancer cells does not correlate with tumour grade (Table III).

**Table II** The relationship between the expression of 5T4 antigen and stage of disease

| Stage of disease | No. examined | 5T4+ (n) Tumour cells and stroma | Stroma only | 5T4− (n) |
|---|---|---|---|---|
| Colorectal carcinoma | | | | |
| A | 8 | 2 | 5 | 1 |
| B | 34 | 7 | 19 | 8 |
| C | 21 | 13 | 7 | 1 |
| D | 9 | 7 | 1 | 1 |
| | | $^{a}P = 0.001$ | | |
| Metastases | | | | |
| negative | 42 | 9 (21%) | 24 | 9 |
| positive | 30 | 20 (67%) | 8 | 2 |
| | | $^{b}P<0.001$ | | |
| Gastric carcinoma | | | | |
| I | 2 | 1 | 1 | – |
| II | 4 | 1 | 2 | 1 |
| III | 1 | 1 | – | – |
| IV | 20 | 12 | 4 | 4 |
| | | [a]NS | | |
| Metastases | | | | |
| negative | 6 | 2 (33%) | 3 | 1 |
| positive | 21 | 13 (62%) | 4 | 4 |
| | | $^{b}P = 0.36$ NS | | |
| All tumours examined | | | | |
| Metastases | | | | |
| negative | 48 | 11 (23%) | 27 | 10 |
| positive | 51 | 33 (65%) | 12 | 6 |
| | | $^{b}P<0.001$ | | |

[a]Comparing stage of cancer for 5T4 positive tumours to stroma positive and negative tumours. [b]Comparing 5T4 positive cancer to stroma positive and negative cancers with or without metastases.

**Table III** Relationship between 5T4 expression and tumour grade

| Histology | No. examined | 5T4+ (n) Tumour cells and stroma | Stroma only | 5T4− (n) |
|---|---|---|---|---|
| Colorectal carcinoma | | | | |
| Well differentiated | 18 | 7 | 8 | 3 |
| Moderately differentiated | 49 | 21 | 22 | 6 |
| Poorly differentiated | 5 | 1 | 2 | 2 |
| | | NS | | |
| Gastric carcinoma | | | | |
| Intestinal | 5 | 2 | 3 | – |
| Mixed | 2 | 1 | 1 | – |
| Diffused | 20 | 12 | 3 | 5 |
| | | NS | | |

NS = not significant.

### Discussion

Previous studies of 5T4 tumour expression have not investigated any possible relationship to tumour stage. A new finding of our study is that the expression of 5T4 antigen in cancer cells correlates with metastatic spread. This association suggests that appearance of this molecule reflects a change in tumour that may contribue to the development of metastatic capacity. The origin of the significant stromal labelling specifically associated with malignancy remains to be determined. It is tempting to speculate that the 5T4 molecules are produced by the tumour cells at low levels and subsequently accumulate in the surrounding stroma in the early stages of cancer.

Other studies have indicated changes in expression of several cell surface glycoproteins in metastasis. Johnson *et al.* (1989) demonstrated that the expression of intracellular-adhesion molecule 1, in human melanoma correlates with increased risk of metastases. Tandon *et al.* (1990) described association between the 323/A3 surface glycoprotein and poor prognosis in breast cancer. These authors found that breast carcinomas larger then 2 cm, without oestrogen receptors, which had involved regional lymph nodes, expressed the 323/A3 antigen more frequently than small, localised and oestrogen receptor positive neoplasms. Investigations on experimentally induced carcinomas have also demonstrated a relationship between tumour progression and a change on the surface of malignant cells (Damen *et al.*, 1991; Nalei *et al.*, 1990; Heffernan *et al.*, 1989; Dennis *et al.*, 1987).

Our results on a positive correlation of the 5T4 expression in cancer cells and their metastatic ability may be important for further therapeutic strategies. The 5T4 mAb is currently being assessed for tumour localisation and may be a useful target in immunotherapy. The ultimate exploitation of the malignancy associated expression of 5T4 molecules in cancer will be influenced by the type of molecule. Using molecular biological approaches to isolate the encoding gene for 5T4 molecules will allow the evaluation of possible relationships to other families of surface molecules such as those with cell adhesion roles.

This work was supported by the Cancer Research Campaign of Great Britain. Dr Starzynska was supported by the Polish Foundation of Gastroenterology.

### References

DAMEN, J.E., SPEARMAN, M.A., GREENBERG, A.H. & WRIGHT, J.A. (1991). Characterization of deoxyguanosine-resistant hypoxanthine-guanine phosphoribosyltransferase (−) metastatic variants altered in soybean-agglutinin-binding properties and cell-surface glycoproteins. *J. Cancer Res. Clin. Oncol.*, **117**, 305–312.

DENNIS, J.W., LAFERTE, S., WAGHORNE, C., BREITMAN, M.L. & KERBEL, R.S. (1987). Beta 1-6 branching of Asn-linked oligosaccharides is directly associated with metastasis. *Science*, **236**, 582–585.

HEFFERNAN, M., YOUSEFI, S. & DENNIS, J.W. (1989). Molecular characterization of P2B/LAMP-1, a major protein target of a metastasis-associated oligosaccharide structure. *Cancer Res.*, 49, 6077–6084.

HOLE, N. & STERN, P.L. (1988). A 72 kD trophoblast antigen defined by a monoclonal antibody. *Br. J. Cancer*, **57**, 239–246.

HOLE, N. & STERN, P.L. (1990). Isolation and characterization of 5T4, a tumour-associated antigen. *Int. J. Cancer*, **45**, 179–184.

JOHNSON, J.P., STADE, B.G., HOLZMAN, B., SCHWABLE, W. & RIETHMÜLLER, G. (1989). *De novo* expression of intercellular adhesion molecule 1 in melanoma correlates with increased risk of metastasis. *Proc. Natl Acad. Sci. USA*, **86**, 641-644.

JONES, H., ROBERTS, G., HOLE, N., MCDICKEN, I.W. & STERN, P.L. (1990). Investigation of expression of 5T4 antigen in cervical cancer. *Br. J. Cancer*, **61**, 96-100.

LOKE, Y.W., WHYTE, A. & DAVIES, S.P. (1980). Differential expression of trophoblast-specific membrane antigens by normal and abnormal human placentae and by neoplasms of trophoblast and non-trophoblastic origin. *Int. J. Cancer*, **25**, 459-461.

MCLAUGHLIN, P.J., CHEN, M.H., SLADE, H.B. & JOHNSON, P.M. (1982). Expression on cultured human tumour cells of placental trophoblast membrane antigens and placental alkaline phosphatase defined by monoclonal antibodies. *Int. J. Cancer*, **30**, 21-26.

NALEI, I.R., WATANABE, H. & RAZ, A. (1990). Identification of B16-F1 melanoma autocrine motility-like factor receptor. *Cancer Res.*, **50**, 409-414.

SOUTHALL, P.J., BOXER, G.M., BASHAWE, K.D., HOLE, N., BROMLEY, M. & STERN, P.L. (1990). Immunohistological distribution of 5T4 antigen in normal and malignant tissues. *Br. J. Cancer*, **61**, 89-95.

TANDON, A.K., CLARK, G.M., CHAMNESS, G.C. & MCGUIRE, W.L. (1990). Association of the 323/A3 surface glycoprotein with tumour characteristics and behaviour in human breast cancer. *Cancer Res.*, **50**, 3317-3321.